# Glucose controls glucagon secretion by directly modulating cAMP in alpha cells

**DOI:** 10.1007/s00125-019-4857-6

**Published:** 2019-04-05

**Authors:** Qian Yu, Hongyan Shuai, Parvin Ahooghalandari, Erik Gylfe, Anders Tengholm

**Affiliations:** Department of Medical Cell Biology, Biomedical Centre, Uppsala University, Box 571, SE-751 23 Uppsala, Sweden

**Keywords:** Ca^2+^, Cyclic AMP, Glucagon release, Hypoglycaemia, Insulin, Pancreatic alpha cell, Protein kinase A, Somatostatin

## Abstract

**Aims/hypothesis:**

Glucagon is critical for normal glucose homeostasis and aberrant secretion of the hormone aggravates dysregulated glucose control in diabetes. However, the mechanisms by which glucose controls glucagon secretion from pancreatic alpha cells remain elusive. The aim of this study was to investigate the role of the intracellular messenger cAMP in alpha-cell-intrinsic glucose regulation of glucagon release.

**Methods:**

Subplasmalemmal cAMP and Ca^2+^ concentrations were recorded in isolated and islet-located alpha cells using fluorescent reporters and total internal reflection microscopy. Glucagon secretion from mouse islets was measured using ELISA.

**Results:**

Glucose induced Ca^2+^-independent alterations of the subplasmalemmal cAMP concentration in alpha cells that correlated with changes in glucagon release. Glucose-lowering-induced stimulation of glucagon secretion thus corresponded to an elevation in cAMP that was independent of paracrine signalling from insulin or somatostatin. Imposed cAMP elevations stimulated glucagon secretion and abolished inhibition by glucose elevation, while protein kinase A inhibition mimicked glucose suppression of glucagon release.

**Conclusions/interpretation:**

Glucose concentrations in the hypoglycaemic range control glucagon secretion by directly modulating the cAMP concentration in alpha cells independently of paracrine influences. These findings define a novel mechanism for glucose regulation of glucagon release that underlies recovery from hypoglycaemia and may be disturbed in diabetes.

**Electronic supplementary material:**

The online version of this article (10.1007/s00125-019-4857-6) contains peer-reviewed but unedited supplementary material, which is available to authorised users.

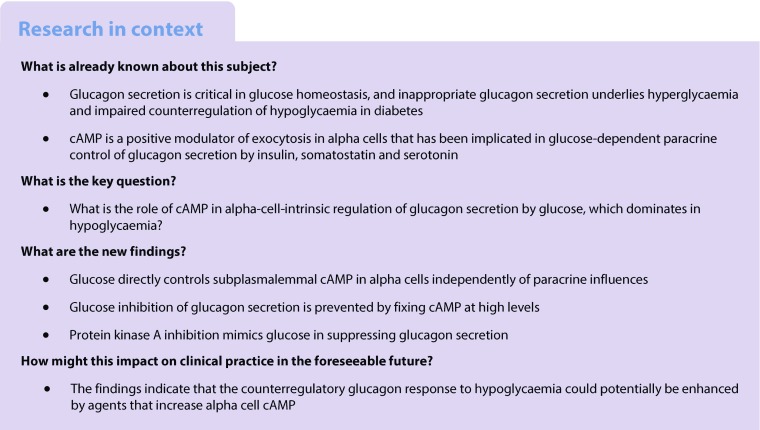



## Introduction

Glucagon is released from pancreatic alpha cells in response to hypoglycaemia, amino acids, adrenaline (epinephrine), other hormones and neurotransmitters [1]. Its main effect is to mobilise glucose from the liver, thereby preventing dangerous reductions in the blood glucose concentration. Individuals with diabetes often show chronically increased glucagon secretion, which contributes to hyperglycaemia [2]. Hyperglucagonaemia may even be more important for the clinical presentation of diabetes than insulin deficiency [3]. Diabetes is also characterised by impaired glucagon secretion in response to hypoglycaemia, which increases the risk of therapy-related glucopenia [4]. Despite the importance of glucagon for normal glucose homeostasis and the aberrant secretion in diabetes, there is limited understanding of the mechanisms by which glucose controls glucagon release [1, 5, 6].

While extra-pancreatic glucose sensors, including neurons in the brain, contribute to the regulation of glucagon secretion in vivo [7, 8], it is evident that glucose is also able to control glucagon release in denervated preparations, such as isolated islets [9, 10]. Such non-neuronal control may be indirect and mediated by glucose-regulated release of paracrine factors from beta and delta cells that influence alpha cell function. Accordingly, insulin, Zn^2+^ and the neurotransmitters serotonin, γ-aminobutyric acid and its metabolite γ-hydroxybutyrate from beta cells have been found to suppress glucagon secretion in some [3, 11–15] but not all [16–18] studies, and somatostatin from delta cells potently inhibits glucagon release [16, 19, 20]. However, a strong argument against the involvement of beta cell factors is that insulin and glucagon secretion are regulated by glucose in different concentration ranges. Glucagon release is stimulated by hypoglycaemia and maximally suppressed at around 7 mmol/l glucose, which corresponds to the threshold for stimulation of insulin secretion in mouse islets [9]. Somatostatin secretion, on the other hand, is stimulated at the low glucose concentrations that control glucagon secretion [9]. However, glucose elevation also inhibits glucagon secretion in islets from somatostatin knockout mice [16] and when somatostatin receptor signalling is inhibited [9, 16, 21], indicating somatostatin-independent effects of the sugar.

There are several hypotheses for how glucose elevation inhibits glucagon secretion by alpha-cell-intrinsic mechanisms. For example, glucose has been found to depolarise alpha cells by ATP-sensitive K^+^ channel closure [10, 18]. The depolarisation causes voltage-dependent inactivation of Na^+^ and Ca^2+^ channels, which reduces Ca^2+^ influx via P/Q-type Ca^2+^ channels that have been claimed to be particularly important for exocytosis [22]. Other studies have instead indicated that glucose reduces Ca^2+^ influx by hyperpolarising the alpha cells after glucose-induced inactivation of a store-operated current [23], by stimulating the Na/K pump [24] or possibly by activating other channels [25, 26]. Although Ca^2+^ is regarded as the main trigger of glucagon exocytosis [27, 28], recordings from alpha cells within intact islets have shown that Ca^2+^ signalling is only modestly or transiently reduced under conditions in which glucose strongly suppresses glucagon release [29], raising the possibility that Ca^2+^ plays a permissive role and that exocytosis is regulated by other factors.

cAMP amplifies Ca^2+^-dependent exocytosis in alpha cells [27], and a reduction in cAMP was recently suggested to account for glucose-induced paracrine inhibition of glucagon release by insulin, somatostatin [30] and serotonin [15]. In the present study, we tested the hypothesis that cAMP mediates alpha-cell-intrinsic glucose sensing. Little is known about cAMP regulation in alpha cells. Using total internal reflection fluorescence (TIRF) microscopy and fluorescent reporters combined with measurements of glucagon release, we investigated how cAMP and Ca^2+^ concentrations in the sub-membrane space ([cAMP]_pm_ and [Ca^2+^]_pm_) of alpha cells change in response to glucose and other modulators of glucagon secretion.

## Methods

### Materials

HEPES, poly-l-lysine, diazoxide and glutamate were obtained from Sigma (St Louis, MO, USA). Penicillin, streptomycin, glutamine and FBS were from Invitrogen (Carlsbad, CA, USA). The somatostatin receptor type 2 (SSTR2) antagonist PRL2903 was provided by DH Coy (Tulane University, New Orleans, LA, USA). Another SSTR2 antagonist, CYN 154806, and pertussis toxin were obtained from Bio-Techne (Abingdon, UK). A cAMP translocation biosensor was generated as previously described [31, 32]. The biosensor encodes a truncated and membrane-anchored protein kinase A (PKA) regulatory RIIβ subunit, unlabelled or tagged with cyan fluorescent protein, and a catalytic Cα subunit tagged with yellow fluorescent protein (YFP) or the red fluorescent protein mCherry. The red version combined with the unlabelled regulatory subunit was used when simultaneously recording Ca^2+^. The fluorescence resonance energy transfer (FRET)-based cAMP sensor Epac-S^H188^ [33] was used for the experiments in Fig. 4. Superfusion and batch incubation of islets were conducted with experimental buffer containing 138 mmol/l NaCl, 4.8 mmol/l KCl, 1.2 mmol/l MgCl_2_, 1.3 or 2.6 (hormone release) mmol/l CaCl_2_, 1–20 mmol/l glucose, 25 mmol/l HEPES and 0.5 mg/ml albumin with pH adjusted to 7.40 with NaOH.

### Pancreatic islet isolation and culture

Islets of Langerhans were isolated from 6- to 9-month old, normal-weight, C57B16J mice of both sexes [9]. The mice were obtained from Taconic (Ry, Denmark) and housed in ventilated cages (up to 5 animals/cage) with a 12 h dark/light cycle and with free access to water and a standard mouse chow. All procedures for animal handling were approved by the Uppsala Animal Ethics Committee. After isolation, the islets were cultured for 1 day in RPMI 1640 medium containing 5.5 mmol/l glucose, 10% FBS, 100 U/ml penicillin and 100 μg/ml streptomycin at 37°C in a 5% CO_2_ humidified air atmosphere. For some experiments, the islets were dispersed to single cells by pipetting in cell dissociation buffer containing 10% (vol./vol.) TrypLE (Thermo Fisher Scientific, Waltham, MA, USA). When all islets were disintegrated, the enzyme digestion was interrupted by adding serum-containing RPMI 1640 medium, followed by centrifugation (5 min at 160 *g*) and resuspension of the cells in the culture medium.

Human islets from three normoglycaemic cadaveric organ donors (two men, one woman, ages 30–72 years) were obtained via the Nordic Network for Clinical Islet Transplantation in Uppsala. All experiments with human islets were approved by the Uppsala Human Ethics Committee. The isolated islets were cultured for up to 7 days at 37°C in an atmosphere of 5% CO_2_ in CMRL 1066 culture medium containing 5.5 mmol/l glucose, 100 U/ml penicillin, 100 μg/ml streptomycin, 2 mmol/l glutamine and 10% FBS.

### Recordings of [cAMP]_pm_ and [Ca^2+^]_pm_

Islets and cells were infected with cAMP biosensor adenoviruses during 1.5 h incubation in 50 μl medium (approximately 10^5^ fluorescence forming units/islet), followed by washing and culture for 16–20 h before use. Islets expressing the cAMP biosensor were preincubated for 20–30 min in experimental buffer. For measurements of [Ca^2+^]_pm_, the preincubation buffer was complemented with 1.2 μmol/l of the acetoxymethyl ester of the Ca^2+^ indicator Fluo-4 (Life Technologies, Carlsbad, CA, USA). After washing in indicator-free buffer, the islets were allowed to attach onto poly-l-lysine coated coverslips during 5–10 min. Coverslips with the islets or cells were used as exchangeable bottoms of an open 50 μl chamber that was superfused with experimental medium (0.12–0.2 ml/min) at 37°C. [cAMP]_pm_ and [Ca^2+^]_pm_ were measured using TIRF or confocal microscopy (see electronic supplementary material [ESM] Methods for details).

### Measurements of glucagon and insulin release

**Fig. 1:**
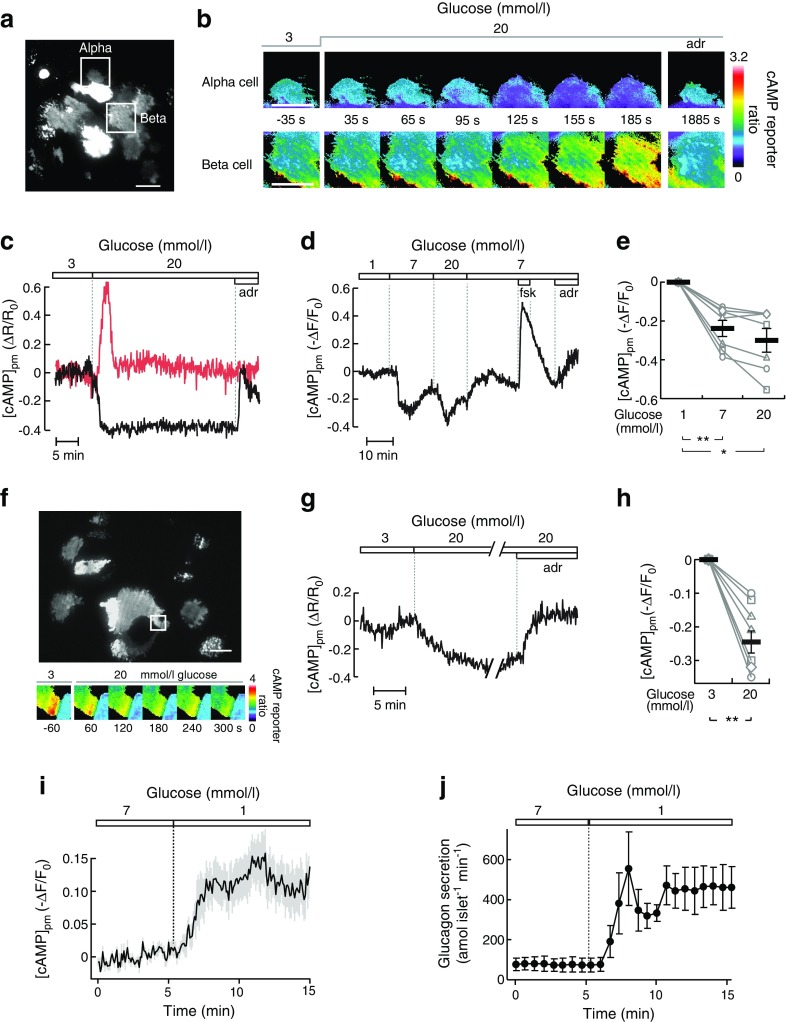
Glucose-induced modulation of [cAMP]_pm_ in alpha cells parallels changes in glucagon secretion. (**a**) TIRF image of the cAMP reporter PKA-Cα-YFP expression in an intact mouse islet. The squares indicate an alpha cell and a beta cell from which [cAMP]_pm_ was recorded. Scale bar, 10 μm. (**b**) Ratiometric TIRF images of the cAMP reporter in the alpha and beta cell highlighted in (**a**). Images were acquired at the indicated times in relation to an increase in the glucose concentration from 3 to 20 mmol/l as well as after applying 10 μmol/l adrenaline (adr). Reductions in the ratio correspond to lowering of [cAMP]_pm_ and vice versa. (**c**) A ratiometric [cAMP]_pm_ recording from the cells highlighted in (**a**) and (**b**). Glucose induced opposite changes in [cAMP]_pm_ in alpha (black line) and beta (red line) cells, identified by their different responses to adrenaline. Changes in the fluorescence ratio (ΔR) have been normalised to the mean ratio during the initial 3 mmol/l glucose exposure (R_0_). Representative of five cells from three independent experiments. (**d**) [cAMP]_pm_ responses in an alpha cell exposed to varying concentrations of glucose, 10 μmol/l forskolin (fsk) and 10 μmol/l adrenaline. The graph represents a single-wavelength recording with the fluorescence intensity changes inverted and normalised to the mean signal during the initial 1 mmol/l glucose exposure. Negative deflections of the curve thus correspond to decreases of [cAMP]_pm_ and vice versa. Representative of seven cells from seven independent experiments. (**e**) Normalised [cAMP]_pm_ levels at increasing glucose concentrations calculated from the seven cells exemplified in (**d**). Data points from each cell are represented with unique symbols connected with lines, together with mean (black horizontal bars) ± SEM for [cAMP]_pm_ at each glucose concentration. (**f**) The large image shows the cAMP reporter PKA-Cα-YFP expression in an intact human islet imaged using TIRF microscopy. Scale bar, 10 μm. The square indicates a region with an alpha cell from which [cAMP]_pm_ was recorded. The montage shows ratiometric TIRF images of the highlighted alpha cell at different time points in relation to an increase in glucose from 3 to 20 mmol/l. (**g**) Ratiometric [cAMP]_pm_ recording from the human alpha cell highlighted in (**f**), showing a glucose-induced reduction and adrenaline-induced elevation of [cAMP]_pm_. After elevation of the glucose concentration, the recording was interrupted for approximately 20 min before the addition of adrenaline (10 μmol/l). Representative of seven alpha cell recordings from three islet donors. (**h**) Quantification of the glucose-induced [cAMP]_pm_ response in seven human alpha cells from the recordings exemplified in (**g**). (**i**) [cAMP]_pm_ response in mouse alpha cells exposed to a decrease in glucose from 7 to 1 mmol/l. The graph represents the mean of nine single-wavelength recordings ± SEM (grey) from four independent experiments, with the fluorescence intensity changes inverted (for positive correlation to [cAMP]_pm_) and normalised to the mean signal during the initial 7 mmol/l glucose exposure. (**j**) Kinetics of glucagon release from batches of perifused mouse islets exposed to a reduction in glucose from 7 to 1 mmol/l. Data are means ± SEM of three recordings from three different islet preparations. Statistical comparisons were made using paired Student’s *t* tests. *p < 0.05, **p < 0.01

The experiments shown in Fig. 5a were performed using a batch incubation protocol with groups of 8–10 islets preincubated for 30 min in experimental medium with 3 mmol/l glucose, followed by 40 min incubation in 500 μl medium containing test substances as previously described [29]. The experiments in Fig. 5b–e and Fig. 6 instead used low-time-resolution perifusion (60 μl/min, 5 min fractions) to eliminate between-group variation (ESM Methods and ESM Fig. 1). Faster perifusion (160 μl/min) and sampling (40–90 s fractions) were used to determine hormone secretion kinetics (Figs 1j and 3f). Groups of 8–10 islets were placed in a closed 10 μl chamber of Teflon tubing with a fine-mesh plastic net at the outlet to prevent the islets from escaping. See ESM Methods for details.

### Quantification and statistical analysis

Image analysis was conducted using MetaFluor (Molecular Devices Corp, Sunnyvale, CA, USA). Fluorescence intensity changes are presented in regions of interest corresponding to single cells with the signal normalised (after background subtraction) to the mean level during the initial condition. In single-wavelength cAMP recordings, the signal was inverted to achieve a positive relationship between normalised intensity and [cAMP]_pm_. In ratiometric cAMP recordings, the ratio changes (positively related to [cAMP]_pm_) were normalised to the initial ratio. Single-cell recordings representative of a given number of cells and experiments (islet donors) are shown, along with quantification of responses as scatter plots or means ± SEM. Statistical comparisons were made using Student’s paired *t* tests.

## Results

### Glucose-induced modulation of [cAMP]_pm_ in alpha cells parallels changes in glucagon secretion

TIRF imaging of mouse islets expressing a cAMP biosensor and exposed to 1–3 mmol/l glucose showed that [cAMP]_pm_ was stable in most cells. An increase in the glucose concentration to 20 mmol/l resulted in decrease of [cAMP]_pm_ in cells identified as alpha cells by their positive [cAMP]_pm_ response to 10 μmol/l adrenaline (Fig. 1a–c). The [cAMP]_pm_ lowering started immediately or after a delay of up to 2.3 min. Half-maximal decrease was observed 1.9 ± 0.2 min after the start of the decline. The beta cells within the same islet usually responded with a [cAMP]_pm_ increase after glucose elevation and often, but not always, with adrenaline-induced lowering (Fig. 1b, c). The effect of 7 mmol/l glucose on alpha cell [cAMP]_pm_ was close to maximal and was often characterised by an initial nadir followed by a somewhat less pronounced sustained reduction (see ESM Results and ESM Fig. 2). Some cells showed additional decrease at 20 mmol/l glucose, but the mean effect did not reach statistical significance (Fig. 1d, e). Alpha cells within human islets showed similar [cAMP]_pm_ reductions in response to glucose elevation (Fig. 1f–h). When the glucose concentration was instead lowered from 7 to 1 mmol/l, mouse alpha cells responded with a rise in [cAMP]_pm_ (Fig. 1i) and perifusion experiments revealed stimulated glucagon secretion with strikingly similar kinetics (Fig. 1j). Control experiments in cAMP biosensor-expressing islet alpha cells loaded with the pH indicator BCECF ascertained that the cAMP responses to glucose did not reflect a pH effect on the biosensor (see ESM Results and ESM Fig. 3).

### Glucose-induced changes in alpha cell [cAMP]_pm_ show little correlation with [Ca^2+^]_pm_

**Fig. 2:**
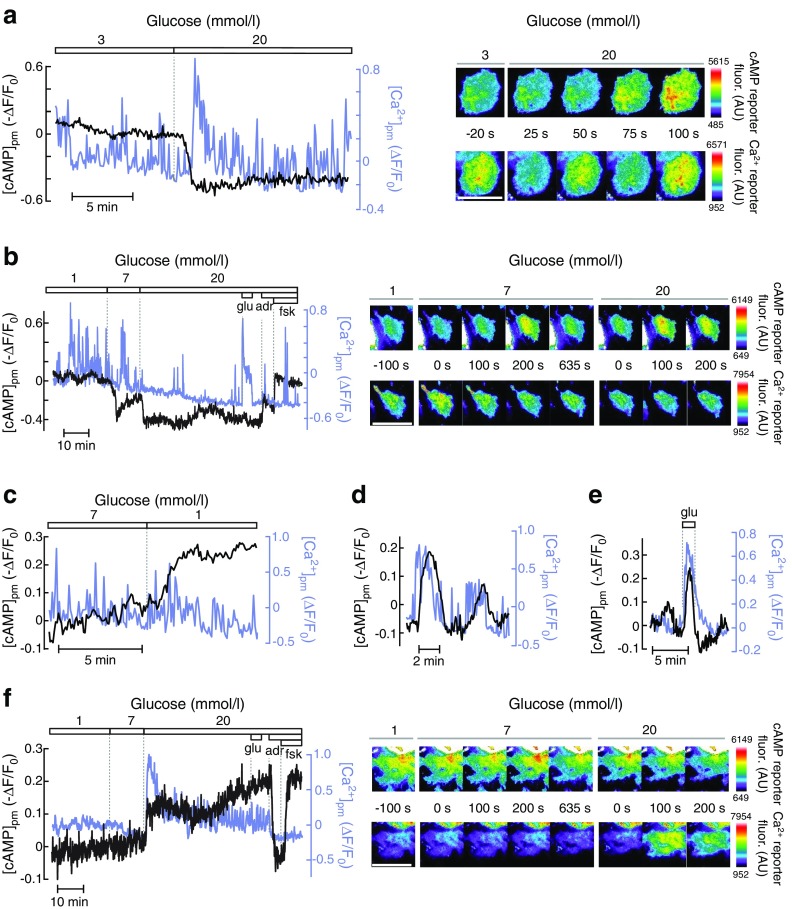
Relationship between [Ca^2+^]_pm_ and [cAMP]_pm_ in alpha cells. (**a**) Simultaneous TIRF recordings of [Ca^2+^]_pm_ (blue line) and [cAMP]_pm_ (black line) in a single alpha cell within an intact mouse islet during elevation of the glucose concentration. The images show the intensities of the fluorescent reporters for cAMP (single-wavelength mCherry version, inversely related to [cAMP]_pm_) and Ca^2+^ (positively related to [Ca^2+^]_pm_) at different time points following an increase in glucose from 3 to 20 mmol/l. The graphs show the intensity changes normalised to the intensity at the initial condition. The cAMP signal has been inverted in the graphs, such that negative deflections of the curve represent decreases of [cAMP]_pm_. Representative of eight cells from six independent experiments. (**b**) Similar [Ca^2+^]_pm_ and [cAMP]_pm_ recording from an alpha cell exposed to varying concentrations of glucose, 1 mmol/l glutamate (glu) and 10 μmol/l each of adrenaline (adr) and forskolin (fsk). The images show the cAMP and Ca^2+^ reporter intensities at different times following the glucose concentration increases. Representative of three cells from three independent experiments. (**c**) [Ca^2+^]_pm_ (blue line) and [cAMP]_pm_ (black line) recordings from an alpha cell exposed to a reduction in glucose from 7 to 1 mmol/l. Representative of nine cells from four independent experiments. (**d**) Example of coordinated oscillatory [Ca^2+^]_pm_ (blue line) and [cAMP]_pm_ (black line) changes in an alpha cell exposed to 7 mmol/l glucose. Representative of two out of 15 cells from eight independent experiments. (**e**) Example of synchronised changes in [Ca^2+^]_pm_ (blue line) and [cAMP]_pm_ (black line) in an alpha cell stimulated by 1 mmol/l glutamate (glu) in the presence of 1 mmol/l glucose. Representative of two out of 48 cells. (**f**) Simultaneous TIRF recordings of [Ca^2+^]_pm_ (blue line) and [cAMP]_pm_ (black line) in a single beta cell within the same islet as the alpha cell shown in (**b**). Glucose induced coordinated increases in [Ca^2+^]_pm_ and [cAMP]_pm_ only when glucose was increased above 7 mmol/l. Representative of seven cells from three independent experiments. Scale bars, 10 μm. AU, arbitrary units

As Ca^2+^ might influence cAMP by regulating adenylyl cyclases and phosphodiesterases, we investigated whether the changes in [cAMP]_pm_ were secondary to those in [Ca^2+^]_pm_ by simultaneously recording the messengers in the same cell. In the presence of 1–3 mmol/l glucose, alpha cells in intact islets typically exhibited fast, irregular [Ca^2+^]_pm_ spiking (Fig. 2a, b). An increase in the glucose concentration to 7 and 20 mmol/l sometimes resulted in a reduced amplitude and frequency of the [Ca^2+^]_pm_ spikes (Fig. 2b) but often lacked a clear effect, or [Ca^2+^]_pm_ even increased, also when [cAMP]_pm_ decreased in the same cell (Fig. 2a). Similarly, when the islets were exposed to a reduction in glucose from 7 to 1 mmol/l, [cAMP]_pm_ increased without a clear effect on [Ca^2+^]_pm_ (Fig. 2c). A link between the two messengers was nevertheless observed in occasional alpha cells. Fig. 2d exemplifies an alpha cell exposed to 7 mmol/l glucose in which slow [Ca^2+^]_pm_ oscillations are accompanied by similar changes in [cAMP]_pm_, and Fig. 2e shows that the alpha-cell-characteristic [Ca^2+^]_pm_ rise in response to glutamate at 1 mmol/l glucose [29] was sometimes associated with an increase in [cAMP]_pm_. In beta cells, [Ca^2+^]_pm_ was low and stable at 1 mmol/l glucose. Elevation to 7 and 20 mmol/l glucose induced an initial lowering in [Ca^2+^]_pm_ followed by concomitant increases in [Ca^2+^]_pm_ and [cAMP]_pm_ (Fig. 2f), consistent with previous observations [34].

### [cAMP]_pm_ lowering by glucose elevation occurs independently of paracrine insulin and somatostatin signalling

**Fig. 3:**
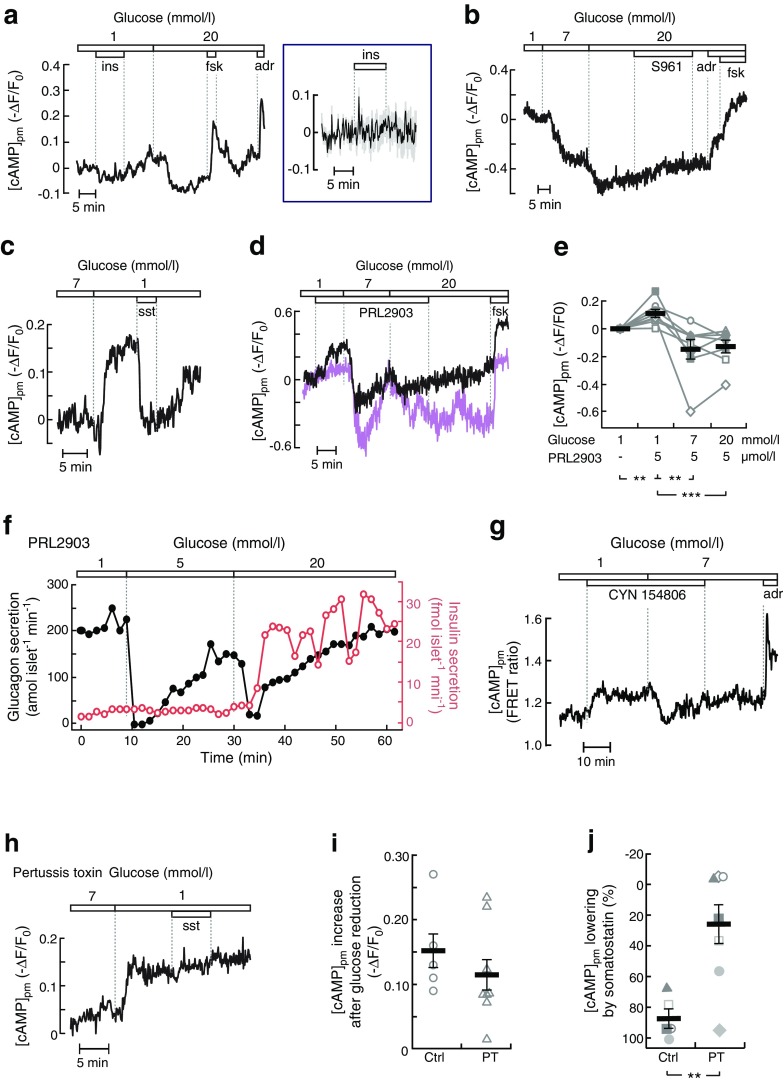
Glucose-induced lowering of [cAMP]_pm_ in alpha cells is not mediated by insulin or somatostatin. (**a**) Effects of 100 nmol/l insulin (ins), 10 μmol/l forskolin (fsk) and 10 μmol/l adrenaline (adr) on [cAMP]_pm_ in an alpha cell within an intact islet exposed to 1 and 20 mmol/l glucose. The box to the right shows the mean ± SEM (grey) for the period of insulin stimulation on an expanded scale (*n* = 15 cells from four independent experiments). (**b**) alpha cell [cAMP]_pm_ recording showing the effects of 1, 7 and 20 mmol/l glucose, 1 μmol/l S961, 10 μmol/l adrenaline and 10 μmol/l forskolin. Representative of ten cells from four independent experiments. (**c**) Effects of reduction in glucose from 7 to 1 mmol/l and addition of 1 nmol/l somatostatin (sst) on [cAMP]_pm_ in an alpha cell within an intact islet. Representative of 13 of 16 cells from six independent experiments. (**d**) Two recordings exemplifying the variability in sensitivity of [cAMP]_pm_ to PRL2903 in the presence of 1 mmol/l glucose. Representative of seven and four alpha cells with (black line) and without (purple line) a PRL2903 effect, respectively, from three independent experiments. Irrespective of the PRL2903 response, increases in the glucose concentration to 7 and 20 mmol/l induced decreases in [cAMP]_pm_. Representative of all of eight cells in three independent experiments testing the effect of glucose. (**e**) Normalised [cAMP]_pm_ levels calculated from eight alpha cells with experimental design as in (**d**). Data points from each cell are represented with unique symbols connected with lines, together with means (black horizontal bars) ± SEM. (**f**) Glucagon (black line) and insulin (red line) release recorded from a batch of perifused mouse islets exposed to different glucose concentrations in the presence of PRL2903. Representative of three independent recordings. (**g**) Effects of the SSTR2 inhibitor CYN 154806 (200 nmol/l) on [cAMP]_pm_ determined with the FRET sensor Epac-S^H188^ in a single alpha cell within an islet. The responses are representative of 19 alpha cells from four independent experiments. (**h**) Alpha cell [cAMP]_pm_ recording showing the effects of a reduction in glucose from 7 to 1 mmol/l and the addition of 1 nmol/l somatostatin to an islet treated for 18 h with 200 ng/ml pertussis toxin. Representative of seven cells from three independent experiments. (**i**) Quantification of [cAMP]_pm_ increase after a reduction in glucose from 7 to 1 mmol/l for control (Ctrl) and pertussis toxin (PT) treated alpha cells. Data points from seven cells are shown, together with the means (black horizontal bars) ± SEM. (**j**) Quantification of [cAMP]_pm_ lowering after addition of somatostatin. The data are expressed as the percentage of the response when changing the glucose concentration from 7 to 1 mmol/l prior to somatostatin addition, with individual data points from seven cells and means (black horizontal bars) ± SEM. Statistical comparisons were made using paired Student’s *t* tests. **p < 0.01, ***p < 0.001

To test if [cAMP]_pm_ is influenced by insulin or somatostatin, the hormones or antagonists of their receptors were added to the islets. Insulin (100 nmol/l) had little effect on alpha cell [cAMP]_pm_ at 1 mmol/l glucose, whereas an increase in glucose to 20 mmol/l significantly reduced [cAMP]_pm_ in the same cell (Fig. 3a). Inhibition of the action of endogenous insulin at 20 mmol/l glucose with the insulin receptor antagonist S961 (1 μmol/l) was also without effect (Fig. 3b), indicating that paracrine insulin signalling unlikely contributes to the [cAMP]_pm_ reduction.

Somatostatin was more efficient and lowered [cAMP]_pm_ at 100 pmol/l in three out of 12 cells from four experiments. Of the remaining alpha cells, seven responded to 1 nmol/l, whereas two cells showed [cAMP]_pm_ reductions only after an increase in somatostatin to 100 nmol/l (data not shown). We therefore used 1 nmol/l somatostatin in the remaining experiments and found that 13 out of 16 alpha cells from six experiments responded at this concentration with a reduction in [cAMP]_pm_ (Fig. 3c). The SSTR2 antagonist PRL2903 (5 μmol/l) often increased [cAMP]_pm_ irrespective of the glucose concentration (7/11 alpha cells in three experiments at 1 mmol/l glucose and 7/9 cells in two experiments at 20 mmol/l glucose), but prevented neither [cAMP]_pm_ lowering by increasing glucose from 1 to 7 and 20 mmol/l (Fig. 3d, e), nor inhibition of glucagon secretion by 5 and 20 mmol/l glucose (Fig. 3f). The experiment in Fig. 3f also shows that glucagon release was suppressed by 5 mmol/l glucose without any simultaneous stimulation of insulin secretion, reinforcing that paracrine signalling from beta cells is not important under those conditions.

Another SSTR2 antagonist, CYN 154806, also increased [cAMP]_pm_ at 1 mmol/l glucose in many alpha cells, but did not prevent [cAMP]_pm_ reduction induced by a subsequent elevation in the glucose concentration (Fig. 3g). To exclude the involvement of somatostatin receptors other than SSTR2, we treated the islets with pertussis toxin (200 ng/ml for 18 h) to inhibit the action of Gαi, which transduces signals from all somatostatin receptor subtypes. Pertussis toxin did not affect the [cAMP]_pm_ increase induced by a reduction in the glucose concentration from 7 to 1 mmol/l, but prevented the [cAMP]_pm_-lowering effect of somatostatin (Fig. 3h–j).

### Glucose-induced cAMP modulation involves an alpha-cell-intrinsic mechanism

**Fig. 4:**
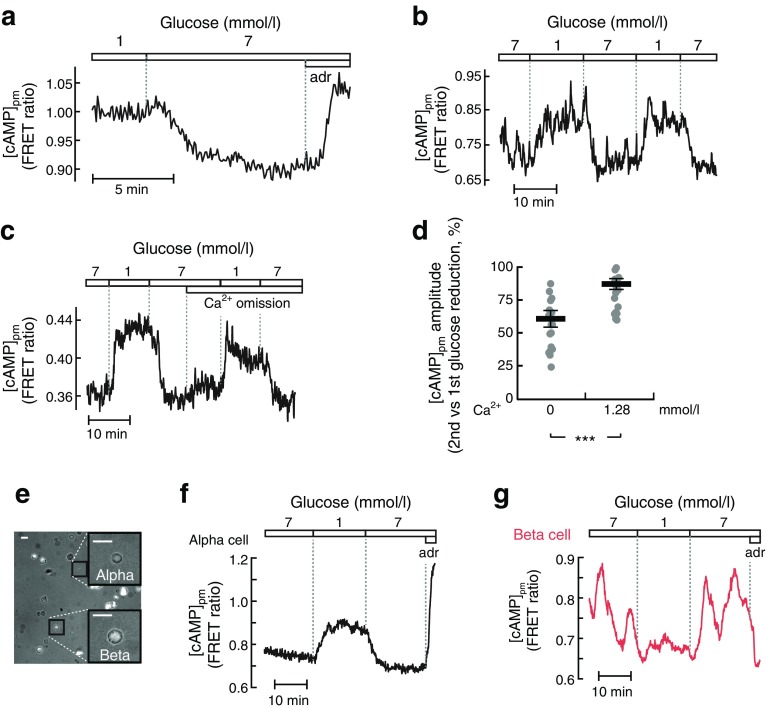
Glucose-induced cAMP modulation involves an alpha-cell-intrinsic mechanism. (**a**) Confocal recording of cytoplasmic [cAMP] in an islet alpha cell expressing the FRET sensor Epac-S^H188^ showing the effect of a change from 1 to 7 mmol/l glucose and addition of 10 μmol/l adrenaline (adr). Representative of five cells from two independent experiments. (**b**) TIRF recording of [cAMP]_pm_ with Epac-S^H188^ during two consecutive decreases of the glucose concentration from 7 to 1 mmol/l. Representative of 22 cells from three independent experiments. (**c**) As in (**b**), but with the omission of Ca^2+^ and addition of 2 mmol/l EGTA after the first glucose reduction. Representative of 20 cells from three experiments. (**d**) Scatter plot with means (black horizontal bars) ± SEM for the amplitude of the [cAMP]_pm_ elevation during the second vs first reduction in glucose from the experiments as shown in (**b**) and (**c**). (**e**) Overlay of transmitted light and TIRF images of dispersed islet cells. Scale bars, 10 μm. (**f**, **g**) TIRF recordings of [cAMP]_pm_ with Epac-S^H188^ from the single, dispersed alpha (**f**) and beta cell (**g**) shown in (**e**) during changes in the glucose concentration and the addition of adrenaline. Representative of 38 (**f**) and 41 (**g**) cells from seven and three independent experiments, respectively. Statistical comparisons were made using paired Student’s *t* tests. ***p < 0.001

To reinforce the conclusion that glucose modulates cAMP in alpha cells, we recorded cAMP with an alternative sensor, the FRET-based Epac-S^H188^ [33]. Confocal detection of the FRET ratio showed that an increase in glucose from 1 to 7 mmol/l triggered lowering of [cAMP] throughout the cytoplasm of islet alpha cells (Fig. 4a). FRET detection with TIRF imaging indicated glucose-induced [cAMP]_pm_ changes similar to those recorded with the translocation sensor, and repeated reductions of glucose from 7 to 1 mmol/l induced recurring [cAMP]_pm_ elevations (Fig. 4b). To consolidate the conclusion that the glucose effect was independent of auto- and paracrine signalling, Ca^2+^ was omitted and 2 mmol/l EGTA added to prevent secretion from all endocrine islet cells. There was a slight increase in [cAMP]_pm_ upon Ca^2+^ removal in the presence of 7 mmol/l glucose (Fig. 4c), possibly reflecting disappearance of paracrine inhibitory signals. Glucose reduction to 1 mmol/l still induced a pronounced [cAMP]_pm_ elevation, although the amplitude was slightly lower than that in the presence of Ca^2+^ (Fig. 4d).

In an additional approach to prevent paracrine signalling, experiments were performed after dispersion of islets into single cells. Individual alpha cells responded to a 7 to 1 mmol/l reduction in glucose or the addition of adrenaline with increases in [cAMP]_pm_ similar to those in islets (Fig. 4e, f). In addition, dispersed beta cells showed islet-similar responses to glucose reduction and adrenaline, with lowering of [cAMP]_pm_ (Fig. 4g).

### Glucose-induced inhibition of glucagon secretion occurs independently of somatostatin and is prevented by fixing cAMP at high levels

**Fig. 5:**
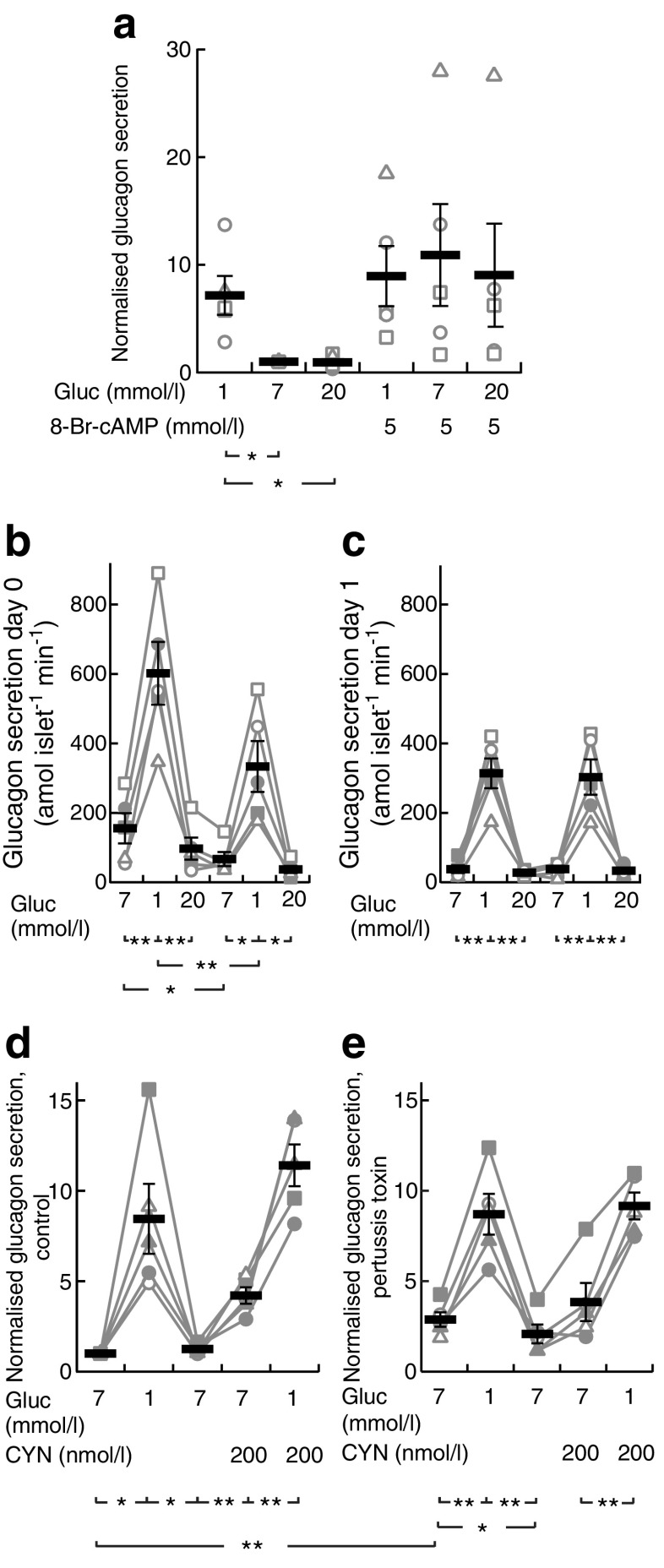
Effects of membrane-permeable cAMP, islet culture and somatostatin signalling on glucose-induced suppression of glucagon secretion. (**a**) Glucagon secretion from batch-incubated mouse islets exposed to different glucose (gluc) concentrations in the absence or presence of 5 mmol/l 8-Br-cAMP. The secretion from each islet batch is normalised to that at the first exposure to 7 mmol/l glucose. Individual data points from five experiments are shown, together with means (black horizontal bars) ± SEM. (**b**, **c**) Glucagon secretion from freshly isolated (day 0, **b**) or overnight cultured (day 1, **c**) mouse islets exposed to sequential changes in glucose concentration. Individual data points from five experiments are shown, together with means (black horizontal bars) ± SEM. (**d**, **e**) Glucagon secretion from islets cultured overnight without (control, **d**) or with 200 ng/ml pertussis toxin (**e**), and subsequently exposed to sequential changes in glucose concentration and 200 nmol/l CYN 154806. The secretion from each batch of islets was first expressed in percent of total glucagon content and then normalised to the initial 7 mmol/l glucose condition in (**d**). Individual data points from five experiments are shown, together with means (black horizontal bars) ± SEM. Statistical comparisons were made using paired Student’s *t* tests. *p < 0.05, **p < 0.01

Next, we investigated how cAMP-modulating agents influence glucagon secretion. In batch-incubated islets, the elevation of glucose from 1 to 7 or 20 mmol/l inhibited glucagon secretion by more than 80%, but in the presence of the membrane-permeable cAMP analogue 8-Br-cAMP secretion remained high independent of the glucose concentration (Fig. 5a). To avoid the variability among groups inherent to batch incubations, we employed an alternative approach based on islet perifusion, allowing sequential exposure of the same islets to different test conditions. This perifusion approach with low temporal resolution was first used to compare the glucagon responses to two successive challenges with 7, 1 and 20 mmol/l glucose on the day of islet isolation (day 0) with islets from the same mouse that had been cultured until the next day (day 1). After 1 day of culture, the glucagon responses to the 1 to 7 mmol/l glucose transitions were more pronounced and reproducible, with approximately eightfold increases compared with four- to fivefold increases on day 0 (Fig. 5b, c). It is possible that higher unstimulated and stimulated secretion on day 0 reflect dysregulated glucagon release after cell perturbation during islet isolation.

**Fig. 6:**
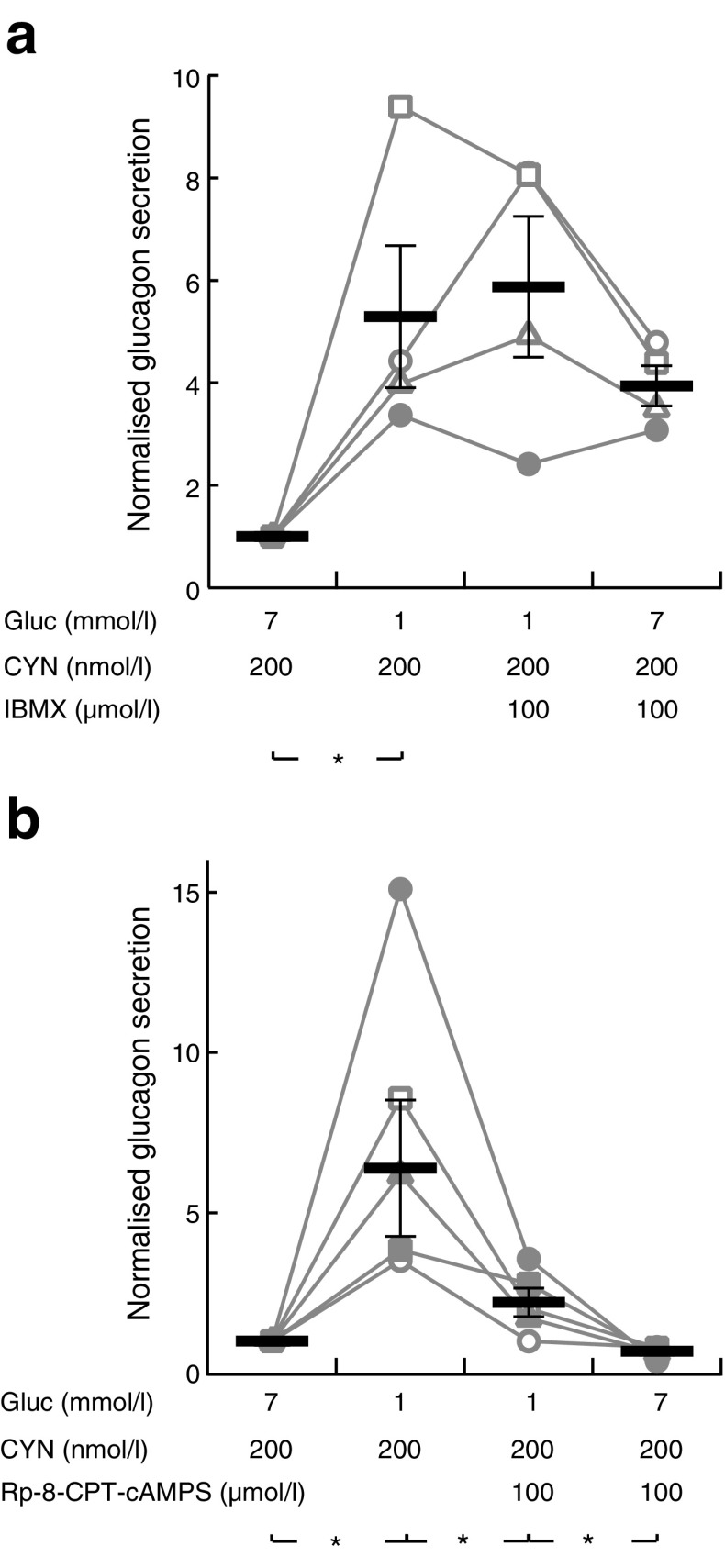
Phosphodiesterase inhibition prevents and PKA inhibition mimics glucose suppression of glucagon secretion. (**a**) Glucagon secretion from mouse islets sequentially exposed to different glucose concentrations and 100 μmol/l IBMX in the continuous presence of 200 nmol/l CYN 154806. Individual data points from four experiments are shown, together with means (black horizontal bars) ± SEM. (**b**) Similar recording showing the effect of different glucose concentrations and 100 μmol/l of the PKA inhibitor Rp-8-CPT-cAMPS in the presence of 200 nmol/l CYN 154806. Individual data points from five experiments are shown, together with means (black horizontal bars) ± SEM, normalised to the initial 7 mmol/l glucose, 200 nmol/l CYN, condition. Statistical comparisons were made using paired Student’s *t* tests. *p < 0.05

Using the perifusion approach with overnight-cultured islets, we investigated how somatostatin influences glucose- and cAMP-regulated glucagon secretion. SSTR2 inhibition with CYN 154806 increased glucagon release at 7 mmol/l glucose but did not prevent 1 mmol/l glucose from inducing a similar increase in secretion as in the absence of the drug (Fig. 5d). After pertussis toxin treatment, the islets showed higher initial glucagon secretion at 7 mmol/l glucose and CYN 154806 lacked a significant effect, but the secretory responses to glucose changes were not different from those of the control (Fig. 5e). Fixing cAMP high with 100 μmol/l of the phosphodiesterase inhibitor 3-isobutyl-1-methylxanthine (IBMX) in the presence of CYN 154806 to evade the influence of somatostatin prevented inhibition of glucagon release in response to a 1 to 7 mmol/l glucose transition (Fig. 6a), underscoring that glucose acts by decreasing cAMP.

### PKA inhibition mimics glucose inhibition of glucagon secretion

Since many effects of cAMP are mediated by PKA, we investigated the potential involvement of this kinase in glucagon secretion. Changing glucose from 7 to 1 mmol/l induced a marked stimulation of glucagon secretion (Fig. 6b). Subsequent introduction of the PKA inhibitor Rp-8-CPT-cAMPS (100 μmol/l) reduced secretion by more than 70%, and there was some further reduction at 7 mmol/l of the sugar (Fig. 6b).

## Discussion

Previous studies have provided evidence that Ca^2+^ is a main trigger of exocytosis in alpha cells [27, 28], and alpha cells show electrical activity and Ca^2+^ signalling in the presence of low glucose concentrations that stimulate glucagon release in hypoglycaemia [27–29, 35]. However, increases in glucose concentration, which strongly inhibit glucagon secretion, have modest or transient effects on alpha cell Ca^2+^ signalling [29, 35]. Therefore, messengers other than Ca^2+^ may be more important in regulating glucagon release. Exocytosis in alpha cells is known to be strongly dependent on cAMP [27]. The present study provides evidence for a key role of cAMP in glucose-regulated glucagon secretion mediated by a direct effect of the sugar on alpha cells.

We found that glucose modulated [cAMP]_pm_ in both mouse and human alpha cells. Since mouse alpha cells have been reported to express glucagon receptors [36], and glucagon, at least in some mouse alpha cells, increases [cAMP]_pm_ [34], glucose-induced decrease of [cAMP]_pm_ might simply be the consequence rather than the cause of inhibited glucagon secretion. Indeed, [cAMP]_pm_ and secretion showed strikingly similar kinetics. However, the glucose-induced changes in alpha cell cAMP persisted when glucagon secretion was inhibited by Ca^2+^-deficient medium. Moreover, the glucose-induced reduction in glucagon secretion was prevented when the intracellular cAMP concentration was fixed at a high level, either by membrane-permeable cAMP or a phosphodiesterase inhibitor. These observations strongly indicate that lowering of [cAMP]_pm_ underlies the suppression of glucagon secretion.

We previously reported that very high glucose concentrations induce increases in [cAMP]_pm_ with oscillations in a small fraction of alpha cells [34]. The responses sometimes involved alternating increases and decreases of [cAMP]_pm_ above and below the baseline, resembling the pattern of glucagon secretion under similar conditions [37]. The reason for the discrepancy with the presently observed [cAMP]_pm_ reduction by glucose is unknown, but the lowering effect is now extensively substantiated.

Based on immunohistochemical detection of cAMP, it has been suggested that glucose elevation induces paracrine lowering of cAMP in alpha cells by stimulating the secretion of somatostatin and insulin [30]. In other cell types, including adipocytes, insulin is known to promote cAMP degradation by activating phosphodiesterase 3B [38]. However, we found that insulin itself or blockade of its receptor lacked an effect on alpha cell cAMP, indicating that paracrine insulin signalling is not involved. Alpha cells also express somatostatin receptors with domination of SSTR2 [39, 40]. Somatostatin receptors activate G_i/o_ to suppress adenylyl cyclase activity, and the present study consequently showed that exogenously applied somatostatin lowers [cAMP]_pm_ in most alpha cells, an effect that was prevented when G_i/o_ signalling was inhibited with pertussis toxin. However, the toxin did not interfere with the glucose reduction-induced increase in [cAMP]_pm_. The SSTR2 antagonist PRL2903 increased [cAMP]_pm_, which indicates that endogenous somatostatin affects alpha cell cAMP, but the drug did not prevent glucose-induced [cAMP]_pm_ reduction. Another SSTR2 inhibitor, CYN 154806, also did not influence the glucose-induced reduction in [cAMP]_pm_, although it strongly increased glucagon secretion. These observations are consistent with previous conclusions that somatostatin has a tonic inhibitory effect on glucagon secretion [9, 16, 21]. More importantly, the data clarify that glucose elevation reduces [cAMP]_pm_ in alpha cells independent of paracrine influences from insulin or somatostatin. It is also unlikely that serotonin [15] or other factors secreted from islet cells mediate cAMP regulation by glucose, since influx of Ca^2+^ is required for exocytosis in all major islet cell types [21, 28, 41] and the effect of glucose was maintained under Ca^2+^-deficient conditions. We therefore conclude that glucose controls cAMP and glucagon release by a direct effect on the alpha cell.

It remains to be elucidated how glucose lowers cAMP. In beta cells, glucose increases [cAMP]_pm_ by activating adenylyl cyclases via elevations of Ca^2+^ [42] and the cAMP precursor ATP [32]. There are also adenylyl cyclases that are inhibited by Ca^2+^ [43] and Ca^2+^-stimulated phosphodiesterases degrading cAMP [44, 45]. Little is known about the adenylyl cyclase and phosphodiesterase protein expression profiles in alpha cells, but several observations indicate that Ca^2+^ does not mediate the effect of glucose on [cAMP]_pm_. Although simultaneous recordings of the two messengers indicated that Ca^2+^ sometimes seems to promote increases in alpha cell [cAMP]_pm_, there was generally no strong correlation between glucose-induced changes in [cAMP]_pm_ and [Ca^2+^]_pm_. Moreover, in accordance with previous studies [29, 35], [Ca^2+^]_pm_ showed modest glucose dependence in alpha cells. Most importantly, the glucose effect on [cAMP]_pm_ remained when Ca^2+^ entry was prevented by Ca^2+^-deficient conditions.

There are alternative hypotheses for the Ca^2+^-independent effects of glucose on [cAMP]_pm_. In beta cells, glucose has been found to activate phosphodiesterase 3B in a Ca^2+^- and insulin-independent manner, associated with changes in enzyme phosphorylation [46]. A similar mechanism may operate in alpha cells. Our previous work has emphasised a role for store-operated Ca^2+^ entry in regulating alpha cell electrical activity, Ca^2+^ signalling and glucagon secretion [9, 23]. Interestingly, it has been suggested that cAMP production could be regulated by a store-operated mechanism independently of Ca^2+^ [47]. Since glucose reduces store-operated signalling in alpha cells [48], such a mechanism can be expected to result in reduced cAMP production.

The link between cAMP and glucagon secretion was studied in islets cultured overnight, which were found to have a more pronounced and consistent secretory response to repeated low-glucose challenges than freshly isolated islets. The cAMP effects seem to involve PKA, since, in line with a previous report [30], inhibition of this kinase suppressed glucagon secretion at 1 mmol/l glucose, whereas another study [49] found no effect of a different PKA inhibitor. cAMP may act in part by activating the voltage-gated Ca^2+^ channels that mediate exocytosis-triggering Ca^2+^ influx [27]. The effects are complex and have been suggested to involve PKA-mediated inhibition of N- [49] or P/Q-type [50] channels at small cAMP elevations, and Epac-dependent stimulation of L-type channels at higher elevations [49]. However, a quantitatively more important effect of cAMP is to accelerate the mobilisation of granules to the readily releasable pool [27], consistent with the poor correlation between alpha cell [cAMP]_pm_ and [Ca^2+^]_pm_ as well as the modest changes in alpha cell [Ca^2+^]_pm_ that accompany glucose-regulated glucagon secretion. Future studies will clarify whether the residual stimulation of glucagon secretion at 1 mmol/l glucose in the presence of PKA inhibitor (Fig. 6b) is mediated by Epac.

We now propose that glucose concentrations in the hypoglycaemic range regulate glucagon secretion by directly modulating the cAMP concentration in alpha cells. Such a mechanism does not exclude a further role for cAMP in the paracrine regulation of glucagon release [15, 30], which may become dominating during hyperglycaemia [5]. Although Ca^2+^ is a critical trigger of exocytosis in alpha cells, the ion seems to have a more permissive role and the magnitude of secretion is controlled mainly by cAMP-mediated amplification of granule exocytosis. Further studies are warranted to clarify the mechanisms by which alpha-cell-intrinsic glucose sensing controls glucagon secretion via cAMP, and whether aberrant cAMP signalling underlies the dysregulated glucagon secretion in diabetes.

## Electronic supplementary material

ESM (PDF 864 kb)

## Data Availability

The datasets generated and/or analysed during the current study are available from the corresponding author on reasonable request.

## Abbreviations

[Ca^2+^]_pm_: Subplasma membrane Ca^2+^ concentration
[cAMP]_pm_: Subplasma membrane cAMP concentration
FRET: Fluorescence resonance energy transfer
IBMX: 3-Isobutyl-1-methylxanthine
PKA: Protein kinase A
SSTR2: Somatostatin receptor type 2
TIRF: Total internal reflection fluorescence
YFP: Yellow fluorescent protein
