# Supplementary material for: Glucose controls glucagon secretion by directly modulating cAMP in alpha cells
Source: Diabetologia. 2019 Apr 5;62(7):1212–24. doi: 10.1007/s00125-019-4857-6 (PMC6560012; doi:10.1007/s00125-019-4857-6)
Supplement: Supplementary file 1 — (PDF 864 kb) [file 125_2019_4857_MOESM1_ESM.pdf]

Electronic Supplementary Material

ESM Methods

Total internal reflection microscopy imaging of $[Ca^{2+}]_{pm}$ and $[cAMP]_{pm}$

A Nikon Eclipse Ti microscope was equipped with an illuminator for total internal reflection fluorescence (TIRF) imaging. Diode or diode-pumped solid-state lasers (Cobolt, Stockholm, Sweden) provided excitation light at 491 nm (for Fluo-4), 445 nm (CFP), 515 nm (YFP) and 561 nm (mCherry). The laser beams were aligned in an in-house built laser combiner before passing a filter wheel with a shutter (Sutter Instruments, Novato, CA) to select the appropriate wavelength. The beam was then fed into a single-mode optical fiber (Oz Optics, Ottawa, Canada) attached to the TIRF illuminator and the light focused onto the back focal plane of a 60x, 1.45-NA objective. The illumination angle was set by the fiber positioning mechanics of the illuminator to achieve total internal reflection. Fluorescence was selected with interference filters (Semrock, Rochester, NY) at 530 nm/50 nm half-bandwidth (Fluo-4), 483/32 (CFP), 542/27 (YFP) or a 620 longpass filter (mCherry) in a filter wheel (Sutter Instruments) and detected with a back-illuminated EMCCD camera (DU897, Andor Technology, Belfast, Northern Ireland, UK) controlled by MetaFluor software (Molecular Devices Corp, Downington, PA). Single wavelength images or CFP/YFP image pairs were acquired every 2-5 s. Epac-S^{H188} was excited at 445 nm with donor emission detected at 483/32 nm and sensitized acceptor emission at 542/27 nm. FRET was recorded as the 483/542 nm fluorescence ratio (FRET ratio).

Confocal microscopy

One series of cAMP-dependent FRET recordings in intact islets was performed with a confocal imaging setup. A Nikon TE-2000 microscope was equipped with a Yokogawa

CSU-10 spinning disk confocal unit. A 442-nm diode laser (Oxxius, Lannion, France) was used for excitation and fluorescence was collected with a 60x, 1.4-NA objective at 560/40 nm and 483/32 nm. The emission wavelengths were selected with interference filters (Semrock) in a fast-changing wheel (Sutter Instruments) mounted in front of a back-illuminated EMCCD camera (iXon DU-888; Andor Technology). Image pairs at 483/560 nm were recorded every 5 s. The focal plane for imaging was selected to be at least one cell diameter above the coverslip to enable imaging of cells without glass contact.

Perifusion experiments for measurements of glucagon and insulin secretion

For low-time resolution perifusion experiments, groups of 8-10 islets were placed in a 10 µl Teflon tube chamber and perifused at a rate of 60 µl/min (Figure 5b-e and Figure 6) using a pressurized air system (AutoMate Scientific, Berkeley, CA). After 30 min of equilibration in experimental buffer with 3 mmol/l glucose followed by 10 min in 1 or 7 mmol/l glucose, the perifusate was collected in 5-min fractions into ice-chilled 96 well non-binding-surface plates (Corning Inc. Kennebunk, ME) using a custom-modified fraction collector while changing the glucose concentration and/or adding test substances. The samples were frozen and kept at -20°C up to 3 days until analyses with glucagon ELISA according to the manufacturer instructions for enhanced sensitivity (Mercodia AB, Uppsala, Sweden). Three fractions collected at each condition were analyzed for glucagon (ESM Fig.1). The results are presented as one data point per condition, which represents the average of the last two fractions at that condition. The first fraction was excluded since it is influenced by the preceding condition. In most cases glucagon secretion from each batch of islets was normalized to that at the first exposure to 7 mmol/l glucose. To determine the kinetics of hormone secretion, the islets

were perifused at 150 µl/min and samples were collected every 40 s (Figure 1j) or 90 s (Figure 3f) and assayed for glucagon as above. In the latter experimental series, insulin release was determined from the same samples using a mouse/rat insulin immunoassay kit (Mesoscale Discovery, Rockville, MD).

ESM Results

7 mmol/l glucose induces sustained lowering of alpha cell cAMP

Glucose transition from 1 to 7 mmol/l typically induced $[cAMP]_{pm}$ changes with an initial nadir followed by sustained lowering (ESM Fig. 2).

Control experiments testing the pH sensitivity of the cAMP biosensor

Islets with cAMP biosensor-expressing alpha cells were loaded with the pH indicator BCECF to record $[cAMP]_{pm}$ and pH_{pm} in parallel. When reducing glucose from 7 to 1 mmol/l, 5 alpha cells in 2 experiments showed cAMP elevation without changes in pH (ESM Fig 3a). Increasing glucose from 3 to 20 mmol/l instead caused lowering of cAMP without change in pH in 4 and with a small acidification in 3 alpha cells in 2 experiments (ESM Fig 3b-c). However, in the latter case the kinetics differed underscoring that changes in pH does not underlie a false cAMP response. Moreover, alkalinisation with NH_4Cl had no consistent effect on the cAMP signal.

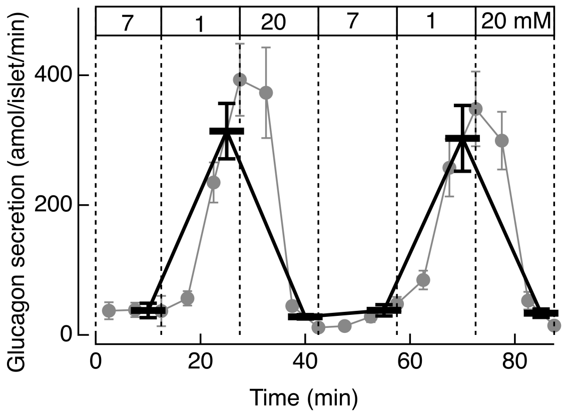

ESM Fig. 1. Kinetics of glucagon release from overnight cultured mouse islets exposed to sequential changes of glucose concentrations. Day 1 secretion data from Fig. 5B (black trace) together with all the underlying data points (gray trace) presented as averages ± s.e.m.

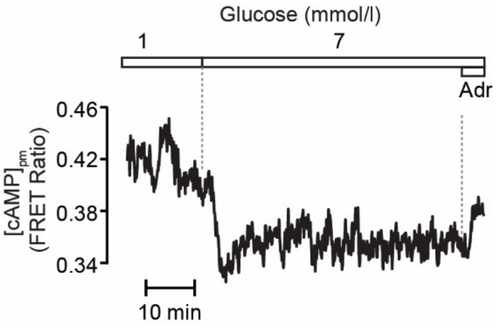

ESM Fig. 2. Glucose induced a sustained lowering of cAMP in alpha cells. cAMP response in an alpha cell exposed to a glucose transition from 1 to 7 mmol/l followed by 10 μmol/l adrenaline. Representative for all of 9 alpha cells in two experiments.

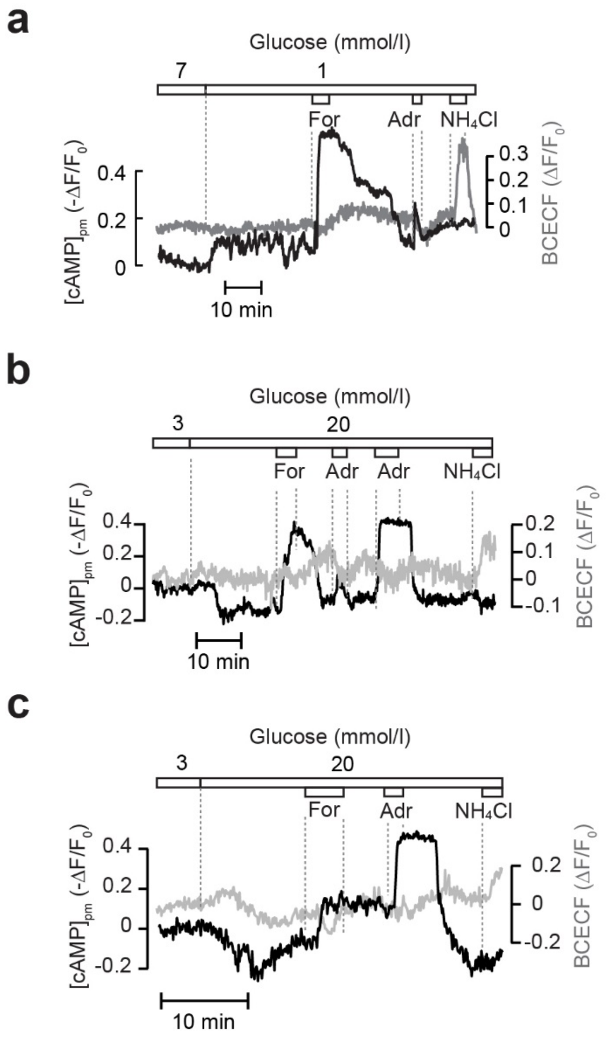

ESM Fig. 3. Analysis of the influence of pH on the translocation cAMP biosensor fluorescence. Effects of varying glucose concentrations, 10 µmol/l forskolin, 10 µmol/l adrenaline and 20 mmol/l NH_4Cl on the fluorescence intensity from the translocation cAMP biosensor and the pH indicator BCECF. (a) Representative for all of 5 alpha cells in two experiments. (b-c) Representative for 4 (b) and 3 (c) of 7 alpha cells in two experiments.
